# The Relationship between Uric Acid and the Development, Complication, and Prognosis of Atrial Fibrillation: The Views from a Clinical Study

**DOI:** 10.1155/2022/9355504

**Published:** 2022-10-27

**Authors:** Jian Yang, Lian Lou, Xuan Zhang, Yuxiao Chen, Weier Zhou, Chi Zhang, Xiaogang Guo, Shenjiang Hu

**Affiliations:** Department of Cardiology, The First Affiliated Hospital, Zhejiang University School of Medicine, Hangzhou, China

## Abstract

A large number of studies suggest that uric acid (UA) is related to the occurrence, complications, and prognosis of atrial fibrillation (AF). However, the guidelines did not clearly elaborate on this issue. The current research results need to be summarized to analyze the association between UA and AF. This study found that in the current clinical research on the relationship between UA and AF, studies mainly focus on the development or complications of AF. A lot of repetitive work does not deepen awareness of this question. In contrast, studies investigating the effects of UA-lowering therapy on the management of AF are limited. The only reports deny the protective effect of UA-lowering therapy. For now, we suggest that UA is close to the occurrence and progression of AF; therefore, it may have important significance as a clinical marker. The role of UA-lowering therapy in the management of AF is one of the next key issues to be explored. It will be a meaningful topic to focus on the latest research on AF ablation and to conduct a secondary analysis to explore the prognostic impact of UA on the latest treatment methods for AF. Multiomics techniques may allow us to have a deeper understanding of the role of UA in AF management in the future.

## 1. Introduction

Atrial fibrillation (AF) is one of the most common arrhythmias. Studies have shown that the occurrence of AF is related to many factors such as age, gender, metabolism, alcohol, and heart failure [1–5]. On the other hand, a large number of studies suggest that uric acid (UA) is related to the occurrence, complications, and prognosis of AF [6–8]. However, the guidelines did not clearly elaborate on this issue [9]. In view of the high incidence of AF and its harm, optimizing the risk assessment and management of AF is an urgent scientific problem that needs to be solved. Whether UA can play a role in the management of AF is still lacking effective clinical evidence. In general, the current research results need to be summarized to analyze and summarize the association between UA and AF. Therefore, this study will summarize the current research status of the relationship between UA and AF, analyze its deficiencies, and discuss the direction and needs of future research.

## 2. The Relationship between UA and the Development of AF

A large amount of literature has suggested an association between UA and AF. Among them, cross-sectional studies occupied the majority. The biggest drawback of cross-sectional studies is that they usually draw conclusions about whether the two are related, but it is difficult to judge the specific causal relationship. Nevertheless, cross-sectional research is an important method to first discover the problem, and its conclusion is worthy of attention.

### 2.1. Population-Based Cross-Sectional Study

An observational study based on physical examination data in Tokyo, Japan included 90,117 samples, and AF was identified in 291 subjects (0.32%). After excluding patients with hypertension, diabetes, dyslipidemia, chronic kidney disease, and hyperuricemia with current medication, the regression analyses were conducted on 49294 patients. The results showed in different models that a higher SUA level is one of the characteristics in the AF group after adjustment for interfering factors (model 1 : 3.803 (2.173–6.654); model 2: OR 1.526 (1.21–1.92); model 3: OR 3.187 (1.806–5.623)), which means hyperuricemia is an independent competing risk factor for AF [10]. A study in Tangshan, China, was based on health checkups and included 8937 people aged 18–82. AF was found in 53 subjects, and logistic regression analysis showed hyperuricemia was significantly associated with AF prevalence in females (OR: 6.366 (2.533–15.871, *P* < 0.001)) but not in males (OR: 1.025 (0.400–2.626), *P*=0.96) [11]. Another study in Tangshan, China restricted the population to diabetic patients and included 9,050 diabetic patients based on physical examination data. The results showed that for diabetic patients, UA is an independent predictor for AF [12]. The Guangzhou Heart Study included 11488 individuals aged 35 or older from urban and rural areas of Guangzhou through randomized multistage cluster sampling and found that for women, hyperuricemia is an independent predictor of nonvalvular AF, while no such correlation was found for men [13]. A study in Chengdu selected 1,038 very elderly people (aged 80 or older) in the general community by a stratified three-stage cluster-sampling design, and the results showed hyperuricemia is associated with the prevalence of AF in the general very elderly [14]. Krittayaphong et al. used a two-stage stratified cluster, proportional to the size of the sampling technique, to analyze patients with hypertension in the records of public hospitals across Thailand. A cross-sectional analysis of 71,440 hypertensive patients showed that elevated UA levels are associated with an increased risk of AF [15]. A Japanese study based on single-center physical examination data included 285,882 individuals and found that SUA is significantly associated with the prevalence of AF (multivariate analyses; men: OR 1.19 (1.14–1.24), women: OR 1.44 (1.34–1.55)). This is the study with the largest sample size [16]. Sun et al. found SUA is positively associated with the prevalence of AF in rural China in the analysis of 11338 people in Liaoning, China [17].

In the above population-based cross-sectional studies, SUA showed a significant positive correlation with the prevalence of AF. One study [13] also found the sex-specificity of this association. These studies have focused on Asia and have conducted analyses based on physical examination data or large-scale sample surveys. Regardless of whether the concerned residents are the general population, diabetic individuals, or elderly individuals, the samples selected in these studies are highly representative, which suggests a correlation between the prevalence of SUA and the prevalence of AF in the Asian population.

### 2.2. Center-Based Cross-Sectional Study

In a center-based cross-sectional study in Tokyo, the sex-specificity of the association between SUA and AF prevalence was reported again. 7,155 patients were analyzed and found that SUA is apparently associated with AF prevalence in women [18]. In addition, Liu et al. confirmed the association of SUA with the prevalence of AF in a single-center analysis of 3489 patients over the age of 40 in Beijing, China [19]. Hu et al. reported that the level of SUA in AF patients is increased but is not an independent predictor for AF in a multicenter study [20].

In addition, although the sample size is small, there are some studies that concern specific populations, which have certain implications. For example, Mantovani et al. found that hyperuricemia increases the risk of AF in patients with type 2 diabetes [21, 22]. Memetoglu et al. showed that SUA level can increase the sensitivity and specificity in predicting AF in patients after coronary artery bypass graft (CABG) operation, another study in Turkey found UA is correlated independently with new-onset AF after primary percutaneous coronary intervention (PCI), and Topolyanskaya et al. in Russia reported this association in patients with coronary heart disease [23–25]. There is also a study based on patients with coronary artery disease in Japan. Murakami et al. elaborated that patients with coronary artery disease are more likely to develop AF when the SUA level is high [26]. In China, Wan et al. showed the occurrence of AF in obstructive sleep apnea patients is strongly related to SUA level [27]. The studies of Liu et al. and Shi et al. have proved the correlation between SUA and AF in the hypertensive population [28, 29]. Finally, this correlation has also been mentioned in patients with ischemic heart failure [30].

In general, the correlation between SUA and AF has been extensively demonstrated in cross-sectional studies worldwide, whether they are population-based studies with a large sample size or studies based on a single-center specific group. In contrast, although population-based studies have higher priority, they often miss some risk factors that seemed ignorable in the analysis, such as obstructive sleep apnea, which is discussed in detail in the center-based studies. Therefore, summarizing these conclusions will help to better understand the correlation between SUA and AF. Another point to note is that the center-based cross-sectional studies, except for two large-scale studies in Asia, which were based on the general population of previous visits, the other large number of center-based studies focused on specific groups. This raises concerns about the potential risk of publication bias in the current reported literature. In fact, single-center observational studies that do not find “interesting phenomena” are often difficult to publish. Therefore, whether researchers deliberately screened the analysis group and how the unreported negative studies will affect people's views on this issue are difficult to examine in depth.

### 2.3. A Case-Control Study

Aghaali et al. used the method of case-control study and also demonstrated that SUA is a risk factor for AF [31]. However, another case-control study of 69 to 138 concluded that there was no significant correlation between SUA and new-onset AF [32].

### 2.4. Cohort Study

The conclusions of the cross-sectional studies are limited to their relevance. Although some cross-sectional studies set the main indicator of observation as new-onset AF, which has a certain suggestive implication for the analysis of causality, it is obviously of greater value for cohort studies to explore whether hyperuricemia causes AF. Here, several cohort studies analyzed this issue.

The atherosclerosis risk in communities (ARIC) study is a community-based cohort study, which included 15,382 samples with a median follow-up of 16.8 years. The results suggested that elevated SUA is associated with an increased risk of AF, particularly among blacks (blacks: HR 1.56 (1.28–1.90); whites: HR 1.05 (0.95–1.11)) and women (women: HR 1.25 (1.08–1.43); men: HR 1.05 (0.94–1.18)) [33]. The study with the largest sample size came from America. A cohort study based on medical insurance data included 1,647,812 people and followed them up for about 6 years. It was found that after multivariable-adjustment, in the elderly who were older than 64, gout doubled the risk of incident AF (HR: 1.92 (1.88–1.96), *P* < 0.0001) [34]. In a study conducted by Kuo et al. based on medical insurance data in Taiwan, China, 126,528 people were followed up for 6.1 ± 2.1 years and showed gout is related to AF [35]. Another study in Taiwan, China, suggested that hyperuricemia is a risk factor for AF [36]. Kawasoe et al. conducted a study with a median follow-up time of 4.1 years among 111,556 people in Japan and found that SUA is significantly associated with AF in women [37]. In addition, large-sample population-based cohort studies in Tangshan (China), Norway, South Korea, American, and the United Kingdom also indicated that hyperuricemia or gout are risk factors for AF [38–42].

Other center-based studies have also reached suggestive conclusions. Peters et al. conducted a follow-up survey in Scotland for 22.6 years. This study showed that SUA is closely related to AF in women [43]. Furthermore, there are also conclusions that hyperuricemia is the risk factor for AF in the elderly (aged 65 and over) with normal blood pressure, in type 2 diabetes patients, and in patients with congestive heart failure [44–46].

As mentioned above, especially in population-based studies, the association of high SUA levels with the incidence of AF has been further demonstrated in cohort studies. The stronger association in female patients is also supported by stronger evidence. Moreover, such cohort studies were distributed in many countries, including Asia, America, and Europe, breaking through the limitations of the Asian population we observed in cross-sectional studies. At this point, clinical evidence can basically confirm that high SUA levels cause a higher incidence of AF, especially in women.

## 3. The Relationship between UA and Complications of AF

Embolic events, including ischemic stroke and peripheral embolic events, are the most common complications of AF that have the greatest impact on prognosis. Regarding the studies on complications of AF, most of them focused on embolic events.

### 3.1. A Cross-Sectional Study

To conduct research on embolic events, it is necessary to prospectively or retrospectively establish a large-scale AF patient cohort and carry out long-term follow-up. Such research is often difficult to complete in a single-center experimental design. However, it is interesting that for AF patients, the emboli of the embolic event mainly originate from thrombi in the left atrium [47]. Therefore, the use of transesophageal echocardiography (TEE) to detect left atrial thrombi (LAT) can provide strong evidence for evaluating the risk of embolic events and also provide a good point of penetration for cross-sectional study design.

Tang et al. have reported a TEE observational study of 1,359 patients with nonvalvular AF, and the results suggest that hyperuricemia is a modest risk factor for LAT, which might refine stratification of LAT in AF patients [48]. Our team has also explored this issue recently. Abnormal uric acid metabolism (abUA), defined as elevated levels of SUA or a history of hyperuricemia/gout, was used as a factor in a retrospective study including 2,246 nonvalvular AF patients. After propensity score matching analysis, we found abUA is an independent risk marker for LAT [49]. Spontaneous echo contrast (SEC) is another main outcome indicator in the TEE examination, which is considered to be a change in the early stage of thrombosis [50]. A study in Guangzhou specifically discussed the impact of SUA on SEC and found that SUA level is an independent risk factor for SEC in patients with nonvalvular fibrillation [51]. Another small observational study in Turkey suggested that increased SUA levels are correlated with decreased left atrial appendage peak flow velocity, which may be one of the potential mechanisms of the high risk of LAT caused by hyperuricemia [52]. In addition to the above studies, there are also some studies that have reported the correlation between SUA and increased embolism risk (LAT, SEC, low peak flow velocity) investigated by the TEE examination [53–55].

TEE-related studies have unique advantages. For example, the TEE examination is considered to be the main method for LAT [56]. Researching LAT is obviously more convenient than studying embolic events that occur during long-term survival. However, TEE is often used for LAT screening before ablation or electrical cardioversion rather than routine examination for all AF patients. This makes the retrospectively enrolled patients have higher specificity, such as patients with strong indications for ablation, but they cannot represent general AF populations. Moreover, in recent years, some centers have reported that the use of TEE before ablation is declining, sometimes by more than 50% [57]. With this in mind, it is worth considering whether the populations of studies, which claim to consecutively include AF patients undergoing TEE, are really consecutive. In fact, few TEE studies report their TEE usage rate in ablation patients.

In a single-center retrospective study conducted by Yang et al. in South Korea, patients with acute ischemic stroke were included, of which 412 had cardioembolic strokes. Analysis of them showed that SUA levels are closely related to the risk of cardioembolic stroke [58]. While Liang et al. discussed the correlation between SUA and left ventricular hypertrophy in AF patients [59], these two cross-sectional studies suggest a potential link between SUA and complications other than TEE abnormalities.

### 3.2. Cohort Study

Two cohort studies focusing on SUA and the complications of AF are both from Taiwan and China. One of them is a large sample study based on the insurance database. The 3.0 ± 2.7 years follow-up results of 7,601 people showed that gout, which requires long-term drug treatment, is an important risk factor for stroke in AF patients [60]. Another study prospectively included 160 patients with persistent AF in a single center with a follow-up period of 22 ± 10 months. The results showed that for AF patients, elevated SUA levels and cardiac events (all-cause mortality and hospitalization for heart failure) are independently related [61].

In general, cohort studies on UA and complications of AF are lacking, and relevant evidence is insufficient. Although the two existing studies have certain suggestive significance, they are both limited to Taiwan, China, and the study design also has shortcomings, such as shorter follow-up periods and higher specificity of enrolled populations. In fact, there is relatively strong evidence that UA levels are related to ischemic stroke [62–64]. However, it is not certain whether this type of stroke is cardioembolic or due to other causes.

## 4. The Relationship between UA and the Prognosis of AF

Ablation therapy is an important management tool for AF. Two studies have evaluated the impact of SUA on the ablation prognosis in AF patients. After a retrospective follow-up of 330 AF patients after catheter ablation for 9.3 ± 3.7 months, He et al. found that increased preoperative SUA levels are associated with a higher rate of recurrence of AF [65]. Canpolat et al. prospectively observed the recurrence after cryoballoon-based catheter ablation. During the follow-up period of 19.2 ± 6.1 months, the elevated preablation SUA levels are associated with a higher rate of AF recurrence [66].

In addition to ablation therapy, anticoagulation therapy is also an important part of the management of AF. However, no studies have explored the prognostic effects of SUA on anticoagulation. Anticoagulation is to prevent the occurrence of embolic events, while in the case of regular use of oral anticoagulants, whether warfarin or nonvitamin K antagonist oral anticoagulants, the incidence of embolic events is very low. Under this premise, to study the difference in embolic events between the hyperuricemia group and the control group, a huge sample size is undoubtedly needed as a basis.

## 5. The Effect of Lowering UA Treatment on AF

As mentioned above, a large number of studies have demonstrated the association between UA and AF. Therefore, whether UA-lowering treatment is beneficial to the management of AF has become an important question.

In an animal study, researchers found that febuxostat can reduce endothelial dysfunction and thrombin-antithrombin complex generation by inhibiting xanthine oxidase-mediated oxidative stress, thereby reducing the risk of LAT formation. In theory, the results of this study support the beneficial effect of febuxostat on the prognosis of AF [67]. However, in a population-based matched-cohort study, Kok et al. compared the effects of taking or not taking allopurinol on cardiovascular outcomes in gout patients. 2483 treated and 2483 nontreated patients were included, with a median follow-up time of 5.25 years for the allopurinol group and 5.04 years for the nonallopurinol group. The results showed the allopurinol group had a modest increase in cardiovascular risk (relative risk, 1.20 (1.08–1.34)), which means allopurinol therapy did not provide cardiovascular protection and also led to adverse outcomes in patients with gout. In subgroup analysis, the higher dose group showed a statistically significant difference in the beneficial cardiovascular protection effect. Nevertheless, because of the comparatively lower numbers of cases in the higher dosage groups, a true benefit from a higher daily dose of allopurinol cannot be completely ruled out. In general, the results did not support the association between allopurinol therapy in gout patients with beneficial future cardiovascular outcomes [68].

## 6. What Does Gene Say?

Although clinical research can directly observe the correlation between factors and diseases in patients, clarification of internal causality is often lacking. Recently, Zhou et al. used a metabolomic approach to conduct a high-throughput analysis of metabolic changes in AF. Pathway enrichment analysis of differentially expressed molecules showed that the onset of AF disrupted the purine metabolism pathway and fatty acid metabolism [69]. This study strongly supports the correlation between UA and AF, although it is still unclear whether the change in the UA pathway causes AF.

A mendelian randomization (MR) study is a method to explore the causal relationship between factors and diseases based on the GWAS dataset and differential expression of SNPs. The MR study analyzed by Hong et al. showed that SUA levels are independently associated with the risk of AF [70]. Another MR study showed that high UA is directly related to adverse cardiovascular outcomes [71].

## 7. Discussion

Current studies have shown that uric acid is related to the occurrence, complications, and prognosis of AF (Table 1). Moreover, in addition to clinical observations, some basic studies and MR analysis also support these conclusions. However, as mentioned in the introduction, the guidelines did not explain the relevant content. In our opinion, despite the strong correlation, based on existing evidence, this phenomenon has a very limited role in the clinical management of AF. The failure of febuxostat treatment to achieve the ideal cardiovascular protective effect may be one of the reasons. In fact, there are few studies that discuss this topic. Therefore, exploring the effect of UA-lowering treatment on AF may be an important research direction in the future, although many observational studies have talked about this point in the discussion. It is worth mentioning that conducting such clinical research may face greater ethical challenges. For example, it is difficult to observe whether a patient with gout is prone to AF without UA-lowering treatment.

On the other hand, the relationship between UA and AF requires further basic study of its mechanism, which may help to find more suitable therapeutic targets to obtain greater conversion value. In some basic studies that have been reported, the relevant mechanisms have been elucidated [72]. Xu et al. conducted a study in rats and found that febuxostat and allopurinol, both xanthine oxidase (XO) inhibitors, could reduce the hypertension-related increase in AF perpetuation by restoring calcium handling and gap junction [73]. An earlier study found that febuxostat can inhibit atrial electrical and structural remodeling of AF by suppressing XO and inhibiting the TGF-*β*1/Smad signaling pathway [74]. In a canine model of AF, febuxostat has also been reported to inhibit new-onset AF, possibly through an antioxidant effect [75]. In addition, a recent study suggested that febuxostat increases the predisposition to ventricular arrhythmia by dysregulating calcium dynamics in human cardiomyocytes, which is puzzling because of heterogeneity between atrial and ventricular myocytes, or was it the heterogeneity between human cells and experimental animals that caused the different results [76]. In short, more basic research is still needed to clarify the impact of the UA metabolism pathway on the development of AF and to propose more effective intervention strategies.

At this stage, we suggest that UA (whether SUA level or history of gout) is closely related to the occurrence and progression of AF. Although individual studies have reported gender and ethnicity specificity, in general, this phenomenon is common existing. Relative to its limited therapeutic value, it may have important significance as a clinical marker. When patients have abnormalities in UA, the management of AF may be more active.

In this review, we pooled previous clinical studies investigating the association between SUA and AF. It was found that a large number of studies have reported the correlation between the two, covering development, complications, treatment, and prognosis. It is worth noting that these existing studies did not make UA an important marker in the management of AF. Recent studies are still repeating this known correlation. A common phenomenon is that researchers select specific groups to replicate this association (e.g., patients with hypertension, diabetes, or other comorbidities), which does not drive a deeper understanding of the correlation between UA and AF.

The role of UA-lowering therapy in the management of AF is one of the next key issues to be explored—a subject that has been discussed in many kinds of literature but has been conducted in few. Although limited studies have denied the benefit of UA-lowering therapy, it is still worth exploring whether pharmacological interventions for different types of AF, different origins of AF, and different degree of hyperuricemia can benefit.

Catheter ablation is a first-line treatment plan for AF. Pulmonary vein ablation, alcohol ablation of marshall vein, box ablation, high-power short-duration ablation, etc. are all research hotspots in the treatment of AF. In numerous relevant RCT studies, how the difference in UA level affects the prognosis of patients will be a very meaningful question. Through secondary analysis and meta-analysis of the data from these RCT studies, more clinically instructive answers may be obtained.

Hyperuricemia/gout is a chronic metabolic disease, and it is necessary to explore whether there are better potential intervention targets and more specific clinical markers to closely link UA and the management of AF. Future research using multiomics techniques may give us the answer.

## Figures and Tables

**Table 1:** Details of the literature.

Title	Type	Area	Population	Sample size	Conclusion
Kuwabara et al. International Journal of Cardiology, 2017 [10]	Single-center cross-sectional study.	Tokyo, Japan.	People underwent annual regular health checkup.	90117 people were included.	Hyperuricemia is an independent competing risk factor for AF.
Chen et al. BMJ Open, 2017 [11]	Population-based cross-sectional study from health checkups.	Tangshan, China.	Residents ≥18 years participated in the study with informed consent.	8937 people.	SUA levels were significantly associated with AF prevalence. Females (but not males) with higher SUA levels had an increased prevalence of AF, suggesting a sex-specific mechanism.
Ding et al. QJM, 2015 [12]	Population-based cross-sectional study from health checkups.	Tangshan, China.	Workers with diabetes from the Kailuan Coal Mine Group Corporation.	9050 people were included, of which 60 had AF.	UA is independent predictive factor of AF in both male and female diabetic patients.
Lin et al. BMJ Open, 2019 [13]	Population-based cross-sectional study using randomized multistage cluster sampling.	Guangdong, China.	Residents aged 35 or older.	A total of 11488 residents were included, of which 4547 had hyperuricemia.	Hyperuricemia was highly prevalent among citizens of southern China and was a predictor of nonvalvular AF among women.
Huang et al. Scientific reports, 2018 [14]	Population-based cross-sectional study by using of a stratified three-stage cluster-sampling design.	Chengdu, China.	Very elderly (≥80 years old) people.	1038 participants were enrolled in analysis (55 had AF).	Hyperuricemia is associated with the prevalence of AF in the general very elderly.
Krittayaphong et al. BMC Cardiovascular Disorders, 2016 [15]	Population-based cross-sectional study using a two-stage tstratified cluster-sampling technique.	Thailand.	Hypertensive patients visiting public hospitals.	71440 patients with hypertension were enrolled.	UA level is one of the factors associated with increased risk of AF.
Kawasoe et al. Circulation Journal, 2016 [16]	Single-center cross-sectional study.	Kagoshima, Japan.	People underwent routine health checkups.	285882 people were included.	The UA level was significantly associated with AF, independently of other cardiovascular risk factors.
Sun et al. BMC Cardiovascular Disorders, 2015 [17]	Population-based cross-sectional study using a multistage, randomly stratified, cluster-sampling scheme.	Liaoning, China.	A representative sample of people ≥35 years of age from rural areas.	A total of 11338 people, of which 104 of 9909 with normal SUA levels had AF, and 35 of 1429 with hyperuricemia had AF.	SUA is positively associated with the prevalence of AF in rural China.
Suzuki et al. Circulation Journal [18]	Single-center cross-sectional study.	Tokyo, Japan.	All patients with SUA measurements.	7155 patients were included.	The SUA level was apparently associated with AF prevalence. The independent association in women might imply some sex-specific mechanisms.
Liu Y, et al. Acta Cardiologica, 2010 [19]	Single-center cross-sectional study.	Beijing, China.	Patients over 40 years.	A total of 3489 patients were included, of which 1253 had AF.	An independent relationship between high SUA and AF was confirmed.
Hu et al. Acta Cardiologica, 2010 [20]	Multicenter cross-sectional study.	Taiwan, China.	Aged 55–80 years, diagnosed with hypertension.	3472 patients, of whom 125 had AF.	The level of UA in AF patients is increased, but UA is not an independent predictor for AF.
Mantovani et al. Journal of endocrinological investigation, 2015 [21]	Single-center cross-sectional study.	Verona, Italy.	Patients with type 2 diabetes, who were discharged from endocrinology.	Of the 842 hospitalized patients included, 243 had hyperuricemia and 91 had AF.	Hyperuricemia is associated with an increased prevalence of AF in hospitalized patients with type 2 diabetes, independently of multiple risk factors and potential confounders.
Mantovani et al. Journal of endocrinological investigation, 2017 [22]	Single-center cross-sectional study.	Verona, Italy.	Outpatients with established T2DM, who regularly attended diabetes clinic.	Of the 245 patients with T2DM, 59 had hyperuricemia.	Hyperuricemia is independently associated with an approximately fourfold increased risk of prevalent paroxysmal AF in patients with T2DM.
Memetoglu et al. European review for medical and pharmacological sciences, 2015 [23]	Single-center cross-sectional study.	Istanbul, Turkey.	Patients undergoing their first CABG surgery.	174 patients were included.	The SUA level can increase the sensitivity and specificity in predicting AF in patients after CABG operation.
Karatas et al. Coronary artery Aisease, 2016 [24]	Single-center cross-sectional study.	Istanbul, Turkey.	Patients who were hospitalized with a diagnosis of STEMI and treated with primary PCI.	A total of 621 patients, of whom 40 were diagnosed with new-onset AF after PCI.	SUA levels were found to be correlated independently with new-onset AF after primary PCI.
Topolyanskaya et al. SN Comprehensive Clinical Medicine, 2020 [25]	Single-center cross-sectional study.	Moscow, Russia.	Patients with coronary heart disease over the age of 75.	312 patients, of whom 123 had hyperuricemia.	The diagnosis of AF was significantly increased in patients with hyperuricemia.
Murakami et al. Open Heart, 2017 [26]	Multicenter cross-sectional study.	Japan.	Patients who have undergone coronary angiography.	A total of 1150 patients were included, of which 574 had coronary heart disease and 576 had no coronary heart disease.	Major risk factors of AF are different in patients with or without coronary artery disease. Patients with coronary artery disease are more likely to develop AF when the SUA level is high.
Wan et al. Archives of Medical Research, 2014 [27]	Single-center cross-sectional study.	Xuzhou, China.	Obstructive sleep apnea patients.	516 patients were included while 106 had AF.	The occurrence of AF in obstructive sleep apnea patients is strongly related to SUA level.
Liu et al. Internal Medicine, 2011 [28]	Single-center cross-sectional study.	Tianjin, China.	Patients with essential hypertension.	451 patients were included while 50 had AF.	SUA levels are associated with AF in hypertensive patients.
Shi D et al. Aging Clinical and Experimental Research, 2016 [29]	Single-center cross-sectional study.	Sichuan, China.	Patients with hypertension.	A total of 268 patients, 132 of whom had AF (48 cases of paroxysmal AF and 84 cases of persistent AF).	In patients with hypertension, the presence of AF was associated with arterial stiffness. SUA levels may reflect mechanisms behind this association.
Tekin et al. Angiology, 2013 [30]	Single-center cross-sectional study.	Turkey.	Patients with ischemic heart failure from the outpatient cardiology clinic.	363 patients were included while 78 had AF.	Patients with AF have significantly higher SUA and this was independently associated with AF in patients with ischemic heart failure.
Aghaali et al. Shiraz E-Med J, 2016 [31]	Single-center case-control study.	Qom, Iran.	Consecutive patients with persistent, permanent, or paroxysmal AF who attended the outpatient cardiology clinic or the emergency department were recruited.	32 patients with AF and 32 healthy controls.	Serum uric acid can be considered a risk factor for AF.
Minami et al. Int Heart J, 2009 [32]	Single-center nested cased-control study based on routine annual medical checkups.	Ishikawa prefecture, Japan.	Cases consisted of male workers newly diagnosed with AF based on ECG. Each of these cases had 3 prior annual checkups during which ECGs were negative for AF. Controls, which were matched for age and time period were randomly selected from those never diagnosed with AF over the same 4 yearly evaluations as cases.	69 cases of new-onset AF and 138 controls were included.	New-onset AF was associated with systolic blood pressure and drinking habits, while uric acid is not an independent risk factor.
Tamariz et al. The American Journal of Cardiology, 2011 [33]	Longitudinal community-based cohort study.	United States.	Participants from atherosclerosis risk in communities (ARIC) study.	15382 adults free of AF were enrolled in the analysis with a median follow-up of 16.8 years.	Elevated SUA is associated with a greater risk of AF, particularly among blacks and women.
Singh et al. RMD Open, 2018 [34]	Cohort study based on data from the 5% random sample of medicare claims.	United States.	People older than 64 and younger than 110.	1647812 were observed from 2006 to 2012.	A diagnosis of gout almost doubled the risk of incident AF in the elderly.
Kuo et al. Sci Rep, 2016 [35]	Cohort study using population-based health databases.	Taiwan, China.	Patients diagnosed with gout for the first time between 2000 and 2007 were enrolled for gout group, and 1 : 1 matched patients for control group.	63264 : 63264 patients were enrolled with the mean follow-up duration as 6.1 ± 2.1 years.	Gout was found to be associated with an increased risk of developing AF after adjusting for potential confounders.
Chao et al. International Journal of Cardiology, 2013 [36]	Health insurance-based cohort study.	Taiwan, China.	Patients with hyperuricemia and without AF. For each study patient, an age-, sex- and underlying disease-matched subject without hyperuricemia and history of AF was selected to be the control.	61262 study patients and 61262 controls were enrolled with a mean follow-up duration of 6.3 ± 3.0 years.	Hyperuricemia may be a novel risk factor for the development of AF.
Kawasoe et al. Circulation Journal, 2018 [37]	Cohort study based on data from annual health checkups.	Kagoshima, Japan.	Subjects who underwent annual health checkups at least twice.	111566 subjects were observed during the median period of 4.1 years.	Higher baseline UA was significantly associated with higher AF incidence in women.
Li et al. Journal of the American Heart Association, 2019 [38]	Community-based cohort study.	Tangshan, China.	All residents in the Kailuan community were invited based on Kailuan I and II studies.	123238 participants were included with median follow-up of 6.7 years (interquartile range: 5.3–8.3 years).	High SUA levels and increases in SUA over time were associated with increased risk of incident AF.
Nyrnes et al. Europace, 2014 [39]	Cohort study based on participants from the Tromso study.	Europe.	White Europeans with 25 years and older.	6308 persons were observed during a mean follow-up of 10.8 years.	Baseline SUA was associated with an increased risk for future AF in both sexes.
Kwon et al. Circulation Journal, 2018 [40]	Cohort study based on health screening program.	Seoul, Korea.	Working individuals who participated annual or biennial health examination based on the Korean industrial safety and health law.	282473 subjects were enrolled with a mean follow-up duration of 5.4 ± 3.6 years.	SUA is significantly and positively associated with incident AF in the Korean general population.
Kim et al. Ann Rheum Dis, 2016 [41]	Cohort study using claims data from United HealthCare.	United States.	Adult patients aged ≥40 years who were diagnosed for gout were eligible for the gout group. Two comparison groups were defined ‘osteoarthritis group' and ‘nongout group'.	70015 gout patients with mean (SD) follow-up time as 2.1 (0.8) years and 210045 osteoarthritis patients with 2.0 (1.8). For the comparison between gout and nongout group, 91976 : 275928 patients with 2.0 (1.8) follow-up time were enrolled.	Gout was associated with a modestly increased risk of incident AF compared with osteoarthritis and nongout after adjusting for other risk factors.
Kuo et al. Rheumatology (Oxford), 2016 [42]	Cohort study based on the clinical practice research data-link (CPRD).	United Kingdom.	All incident gout patients diagnosed between 1997 and 2005, and 1 : 1 matched control patients.	90756 patients with a median follow-up of 9 years.	Gout is independently associated with a higher risk of AF at diagnosis and the risk is also higher after the diagnosis.
Peters et al. Heart, 2019 [43]	Cohort study based on multiple overlapping studies.	Scotland.	Participants from Scottish Heart Health Extended Cohort.	15737 participants with a median follow-up of 22.6 years.	UA is strongly associated with the risk of AF in women.
Chuang et al. Nutr Metab Cardiovasc Dis, 2014 [44]	Cohort study based on the elderly nutrition and health survey in taiwan.	Taiwan, China.	People aged 65 years and over in Taiwan.	1485 people were included and the median follow-up time was 9.16 years.	Hyperuricemia is associated with the development of AF in older persons with normal blood pressure.
Valbusa et al. Am J Cardiol, 2013 [45]	Single-center cohort study.	Verona, Italy.	Patients with type 2 diabetes.	400 Caucasian patients with followed for 10 years.	Elevated SUA levels are strongly associated with an increased incidence of AF in patients with type 2 diabetes mellitus even after adjustment for multiple clinical risk factors for AF.
Zhao et al. Chin Med J (Engl), 2012 [46]	Multicenter cohort study.	Hubei, China.	Patients with chronic systolic heart failure.	20259 patients were enrolled with an undeclared follow-up period.	Higher SUA level is associated strongly with AF in patients with chronic heart failure. SUA level can increase int sensitivity and specificity in predicting AF.
Tang et al. Can J Cardiol, 2014 [48]	Single-center cross-sectional study.	Beijing, China.	Consecutive AF patients underwent TEE before the first-time ablation were included.	1359 patients were included and 61 had LAT and another 50 patients had spontaneous echocardiographic contrast (SEC).	Hyperuricemia was a modest risk factor for LAT, which might refine stratification of LAT in patients with nonvalvular AF.
Zhang et al. Eur J Prev Cardiol, 2020 [49]	Single-center cross-sectional study.	Hangzhou, China.	Consecutive patients with nonvalvular AF who underwent transesophageal echocardiography (TEE) before ablation.	2246 patients were included in the study and 30 of them had left atrial thrombosis (LAT).	Abnormal uric acid metabolism is an independent risk marker for LAT.
Liao et al. J Geriatr Cardiol, 2015 [51]	Single-center cross-sectional study.	Guangdong, China.	Consecutive hospitalized AF patients who underwent TEE prior to ablation.	1419 patients with nonvalvular AF were included. 65 had LAT and 57 had SEC.	SUA level is an independent risk factor and has a moderate predictive value for LA-SEC among nonvalvular AF patients in southern China.
Celik et al. Med Princ Pract, 2015 [52]	Single-center cross-sectional study.	Turkey.	Patients with AF, who were admitted to the department of cardiology and in whom TEE was performed before cardioversion, were retrospectively screened.	153 patients were included. Patients with a low left atrial appendage (LAA) peak flow velocity were identified as group 1, and patients with a normal LAA peak flow velocity were identified as group 2.	High SUA levels are associated with a low contractile function of the LAA and could provide additional prognostic information on future thromboembolic events in patients with AF.
Liu et al. Int J Cardiol, 2018 [53]	Single-center cross-sectional study.	Guangdong, China.	Consecutive hospitalized AF patients who underwent TEE prior to ablation.	1198 patients with nonvalvular AF were included. 49 had LAT and 49 had SEC.	Hyperuricemia might independently predict and refine LA stasis risk among nonvalvular patients, especially in those with CHA2DS2-VASc score < 2.
Numa et al. Circ J, 2014 [54]	Single-center cross-sectional study.	Japan.	Consecutive patients with nonvalvular AF who underwent TEE to determine their potential embolic risk.	470 patients were enrolled.	The SUA level was associated with thromboembolic risk on TEE in patients with nonvalvular AF at low-intermediate risk stratified by clinical risk factors.
Ning et al. Int Heart J, 2017 [55]	Single-center cross-sectional study.	Liaoning, China.	Consecutive patients with NVAF who have not accepted anticoagulant therapy.	The study population consisted of 284 patients, and 61 had LAT/SEC.	SUA and left atrial diameter enhanced the predictive ability of CHADS2 and CHA2DS2-VASc for LAT/SEC as additional factors. For patients in moderate risk group, is SUA or LAD was higher than cut-off values, the risk of thromboembolism events would rise accompanied by the elevated risk of LAT/SEC.
Yang et al. J Neurol Sci, 2016 [58]	Single-center cross-sectional study.	Korea.	Consecutive acute ischemic stroke patients.	1489 patients were included. 412 of them were cardioembolic stroke (CES) and 268 of 412 had AF.	SUA level is associated with the risk of CES in acute ischemic stroke patients of both sexes.
Liang et al. Nutr Metab Cardiovasc Dis, 2016 [59]	Single-center cross-sectional study.	Beijing, China.	Consecutive elderly patients (age>60) with nonvalvular AF.	577 patients were included, and 168 of them had left ventricular hypertrophy (LVH).	SUA was independently associated with LVH in elderly male patients with nonvalvular AF.
Chao et al. Int J Cardiol, 2014 [60]	Cohort study based on health insurance database.	Taiwan, China.	Patients 18 years of age or older with newly diagnosed AF were included. Patients who received antithrombotic therapy with either antiplatelet agents or oral anticoagulants were excluded.	A total of 7601 patients who were selected as the study population. The mean follow-up duration was 3.0 ± 2.7 years, and there were 1116 patients who experienced ischemic stroke with an annual stroke rate of around 4.9%.	Hyperuricemia was a significant risk factor of stroke which could potentially refine the clinical risk stratification in AF.
Su et al. Int J Cardiol, 2014 [61]	Single-center prospective cohort study.	Taiwan, China.	Patients with persistent AF referred for echocardiographic examination were enrolled.	160 AF patients were included. The follow-up period to cardiac events was 22 ± 10 months. 40 cardiac events were documented during the follow-up period.	Hyperuricemia was independently associated with an increase in adverse cardiac events and could add significant incremental prognostic value beyond conventional clinical and echocardiographic parameters. Hence, SUA should be measured in AF patients for additional prognostication.
He et al. Chin Med J (Engl), 2013 [65]	Single-center retrospective cohort study.	Beijing, China.	Consecutive patients underwent pulmonary vein isolation as a treatment for paroxysmal AF.	303 patients diagnosed with paroxysmal AF with a mean follow-up of 9.341 ± 3.667 months were analyzed.	In this retrospective study of patients with paroxysmal AF undergoing catheter ablation, elevated preoperative SUA levels were associated with a higher rate of recurrence of AF.
Canpolat et al. Europace, 2014 [66]	Single-center prospective cohort study.	Ankara, Turkey.	Consecutive patients who underwent pulmonary vein isolation with cryoballoon technique for documented AF.	363 patients were enrolled. After a mean follow-up period of 19.2 ± 6.1 months, early recurrence was developed in 33 patients and recurrence after blanking period was observed in 68 patients.	In this prospective study of patients with paroxysmal AF undergoing cryoablation, increased preablation SUA levels were associated with a higher rate of AF recurrence.
Kok et al. PLoS One, 2014 [68]	Population-based retrospective matched-cohort study using health insurance database.	Taiwan, China.	Every individual diagnosed with gout in the years 1999 to 2008 were included. Patients with or without allopurinol treatment were divided and matched.	2483 treated and 2483 nontreated patients were included. The median follow-up time for the allopurinol group was 5.25 years, whereas it was 5.04 years for the nonallopurinol group.	The current population-based matched-cohort study did not support the association between allopurinol therapy in gout patients with normal risk for cardiovascular sequels and beneficial future cardiovascular outcomes. Several important risk factors for cardiovascular diseases, such as smoking, alcohol consumption, body mass index, and blood pressure, were not obtainable in the current retrospective cohort study, which could potentially bias the effect estimate.

## Abbreviations

UA: Uric acid
AF: Atrial fibrillation
OR: Odds ratio
SUA: Serum uric acid
CABG: Coronary artery bypass graft
PCI: Percutaneous coronary intervention
ARIC: Atherosclerosis risk in communities
HR: Hazard ratio
TEE: Transesophageal echocardiography
LAT: Left atrial thrombi
abUA: Abnormal uric acid metabolism
SEC: Spontaneous echo contrast
XO: Xanthine oxidase.
